# Telomerase activity is frequently found in metaplastic and malignant human nasopharyngeal tissues

**DOI:** 10.1054/bjoc.2000.1194

**Published:** 2000-05-18

**Authors:** J T-C Chang, C-T Liao, S-M Jung, T-C V Wang, L-C See, A-J Cheng

**Affiliations:** Department of Radiation Oncology, Department of Otorhinolaryngology, Head and Neck Surgery, Department of Pathology, Chang Guang Memorial Hospital; Department of Molecular and Cellular Biology, Department of Public Health, School of Medical Technology, Chang Gung University, Taoyuan, 333, Taiwan

**Keywords:** telomerase, nasopharyngeal carcinoma, hyperplasia, metaplasia

## Abstract

Telomerase is a specialized ribonucleoprotein polymerase that directs the synthesis of telomere repeats at chromosome ends. Accumulating evidence has indicated that telomerase is stringently repressed in normal human somatic tissues but reactivated in cancers and immortal cells, suggesting that reactivation of telomerase plays an important role in carcinogenesis. In this study, the status of telomerase activity in diseased human nasopharyngeal lesions was determined by the telomeric repeat amplification protocol (TRAP). Fifty-four patients participated including 17 inflammation or hyperplasia, eight with squamous metaplasia, and 29 with different stages of carcinomas. Telomerase activity was detected in 1 of 17 (5.9%) inflammatory or lymphoid hyperplastic tissues, 3 of 8 (37.5%) squamous metaplastic, and 25 of 29 (86.2%) carcinoma tissues. The differences in telomerase expression in these groups is statistically significant (*P*< 0.001). Levels of telomerase activity correlated with tumour stage (*P*= 0.024). These results suggest that telomerase reactivation plays a role in the carcinogenesis of nasopharyngeal cancer. Since telomerase activity is found in the majority of nasopharyngeal cancers and a subset of metaplasia, this enzyme may be served as a reference to monitoring the status of abnormal nasopharyngeal tissues. © 2000 Cancer Research Campaign


*British Journal of Cancer* (2000) **82**(12), 1946–1951
© 2000 Cancer Research Campaign
doi: 10.1054/ bjoc.2000.1194, available online at http://www.idealibrary.com on IDEAL®

### Telomerase activity is frequently found in metaplastic and malignant human nasopharyngeal tissues

**JT-C Chang[1], C-T Liao[2], S-M Jung[3], T-CV Wang[4], L-C See[5] and A-J Cheng[6]**

[1]Department of Radiation Oncology; [2]Department of Otorhinolaryngology, Head and Neck Surgery; [3]Department of Pathology, Chang Guang Memorial Hospital; and [4]Department of Molecular and Cellular Biology; [5]Department of Public Health; [6]School of Medical Technology, Chang Gung University, Taoyuan 333, Taiwan

**Summary** Telomerase is a specialized ribonucleoprotein polymerase that directs the synthesis of telomere repeats at chromosome ends. Accumulating evidence has indicated that telomerase is stringently repressed in normal human somatic tissues but reactivated in cancers and immortal cells, suggesting that reactivation of telomerase plays an important role in carcinogenesis. In this study, the status of telomerase activity in diseased human nasopharyngeal lesions was determined by the telomeric repeat amplification protocol (TRAP). Fifty-four patients participated including 17 inflammation or hyperplasia, eight with squamous metaplasia, and 29 with different stages of carcinomas. Telomerase activity was detected in 1 of 17 (5.9%) inflammatory or lymphoid hyperplastic tissues, 3 of 8 (37.5%) squamous metaplastic, and 25 of 29 (86.2%) carcinoma tissues. The differences in telomerase expression in these groups is statistically significant ($P < 0.001$). Levels of telomerase activity correlated with tumour stage ($P = 0.024$). These results suggest that telomerase reactivation plays a role in the carcinogenesis of nasopharyngeal cancer. Since telomerase activity is found in the majority of nasopharyngeal cancers and a subset of metaplasia, this enzyme may be served as a reference to monitoring the status of abnormal nasopharyngeal tissues. © 2000 Cancer Research Campaign

**Keywords:** telomerase; nasopharyngeal carcinoma; hyperplasia; metaplasia

Nasopharyngeal carcinoma (NPC) is a unique cancer. It is a tumour of epidermoid origin with an endemic distribution among well-defined ethnic groups, present in several world regions. Southeastern China and Taiwan have the highest incidence (10–80 per 100 000 persons per year), followed by North Africa, the Philippines, and the Caribbean nations, but the tumour is rare in Caucasians (Yu, 1991; Fandi and Cvitkovic, 1994; 1995). NPC occurs more frequently in males, at a rate approximately 3-fold that in females (Fandi and Cvitkovic, 1995; 1994). Aside from the striking racial predilection of the disease, other factors implicated in the development of NPC are the Epstein-Barr virus (EBV), genetic and environmental factors. EBV DNA is present in almost every NPC specimen, and the number of EBV genomic copies is proportional to the degree of histological differentiation (Liebowitz, 1994). However, there is compelling evidence showing that the virus causes a subclinical infection early in life, resulting in over 90% seropositivity of susceptible individuals by 3 years-of-age in the Far East. These facts indicate that EBV may be an essential but not a complete factor for the development of NPC (Choi et al, 1993). The fact that populations in southeastern China and Taiwan are preferentially affected by NPC suggests a genetic predisposition for the disease. Several studies of genetic systems and karyotypes have reported that HLA-2 and HLA-BS, as well as the deletion of chromosomes at the region of 3p14–21, 3p25 and 9p21–25, are often associated with NPC (Lu et al, 1990; Huang et al, 1991; 1994). Although these studies have suggested possible sources of carcinogenesis, little is known about the malignant progression of NPC. Clinically, NPC is a malignancy that is difficult to detect in the very early stage, because there are minimal symptoms and little discomfort at that point. The prognosis is very good if the cancer is diagnosed and treated in the early stages (Chang et al, 1996). However, by the time the disease is symptomatically apparent, it has often progressed to late stage. As with all cancers, the best way to improve patient survival is early detection. A better understanding of the carcinogenesis of NPC may result in the discovery of a diagnostic marker that would allow early detection and management, and thus a better prognosis.

Telomerase is a specialized reverse transcriptase that direct the synthesis of telomeric DNA onto chromosomal ends using a segment of its integral RNA component as a template (Blackburn, 1992). In most human somatic cells, telomerase activity is undetectable and telomeric length is progressively shortened during cell proliferation. Cell senescence is thought to occur when the telomeric length is shortened to a critical point (Campisi, 1997; Morin, 1997). Conversely, immortalized human cells exhibit stabilized telomeric lengths and are positive for telomerase activity (Morin, 1997; Wright et al, 1996). Accumulating evidence indicates that telomerase activity is frequently detectable in primary tumour specimens from malignancies of diverse tissue origins, less frequently in premalignant and benign proliferative tissues, and rarely or not at all in normal somatic tissues (Shay and Bacchetti, 1997). Apparently, activation of telomerase expression plays an important role during carcinogenesis.

We designed this study to investigate telomerase activity in diseased nasopharyngeal tissues, including hyperplastic, metaplastic and malignant tissues. Hyperplasia indicates that cells are proliferating rapidly, while metaplasia indicates that normal differentiated cells are replaced by other differentiated cell types. Both hyperplasia

*Received 18 June 1999*
*Revised 18 February 2000*
*Accepted 24 February 2000*

*Correspondence to:* A-J Cheng and metaplasia are usually found in diseased nasopharyngeal tissues (Feng and Liu, 1991; Kristensen et al, 1989; Zong and Zheng 1989; Kieserman and Stern, 1995). The aims of this study were to investigate the level of telomerase activity in different types of nasopharyngeal diseased tissues, and to correlate the levels of telomerase activity with clinicopathological variables in carcinomas.

### MATERIALS AND METHODS

### Patients, tissues and cells

Biopsies from 54 consecutive patients visiting Otorhinolaryngology or Head and Neck Surgery clinics at Chang Gung Memorial Hospital (Taoyuan, Taiwan) were obtained for study. Written informed consent was obtained from all patients participating in the study. One part of each tissue was examined histopathologically, and the rest was stored in liquid nitrogen until use for telomerase activity assay. All cancers were histologically graded according to the World Health Organization (WHO) classification (Hedinger et al, 1989). For each tissue, the presence of lymphocytes, germinal centres, mucus cells, vessel hyperplasia or metaplasia, was specifically recorded. Samples included 17 with inflammation or hyperplasia, eight with squamous metaplasia and 29 with different stages of carcinomas. Immature squamous metaplasia represents the morphologic epithelial changes from a single or multiple layers of reserve cells to an epithelium composed of three or more layers of cells with features of mature nonkeratinizing squamous epithelial cells (Yeldandi et al, 1996). Tumour staging was classified by the UICC system (Hermanek et al, 1997). Nasopharyngeal cancer-derived cell line NPC076 (Ku et al, 1997), which was used as positive control in the present study, was kindly provided by Dr I Chang from the department of microbiology and immunology in our institute. NPC076 cells were grown at 37°C, 5% $CO_2$ in Dulbecco's modified Eagle's medium (DMEM) containing 10% fetal bovine serum and antibiotics (100 U $ml^{-1}$ penicillin, 100 U $ml^{-1}$ streptomycin and 0.25 $\mu g\ ml^{-1}$ amphotericin B).

### Extraction of cellular proteins

Telomerase activity of tissue samples was assayed blindly with a code number and the results were decoded later. Tissue samples (~50 mg) were homogenized in 300 µl of a lysis buffer (10 mM Tris-HCI, pH 7.5, 1 mM $MgCl_2$, 1 mM EGTA, 0.5% CHAPS (Pharmacia), 10% glycerol, 5 mM mercaptoethanol and 0.1 mM phenylmethylsulfonyl fluoride (PMSF)) in Kontes tubes with matching pestles rotated at 450 rpm. For control cells of NPC076 protein extraction, the number of cells was counted by hemocytometer and resuspended at 3000 cells $\mu l^{-1}$ of the lysis buffer. After 30 min at 4°C, the lysate was centrifuged at 15 000 rpm for 30 min at 4°C. The supernatants of tissue and NPC cell extracts were transferred and prepared for telomerase activity assay. The supernatant of NPC cells was diluted serially to obtain 300, 30 and 3 cells $\mu l^{-1}$, respectively. The protein concentration of each tissue sample was determined using Coomassie protein assay reagent (Pierce).

### Telomerase activity assay

Telomerase activity was assayed by the modified telomeric repeat amplification protocol (TRAP) (Cheng et al, 1997; 1999). At a proper buffered condition, the telomeric repeats were synthesized in the presence of TS primer and enzyme telomerase, which was present in the cellular protein extract. Telomere products were subsequently amplified by PCR reaction in the presence of reverse CX primer. Briefly, 0.3 µg of extract protein was added to a 30 µl reaction mixture containing 0.1 µg [$\gamma$-$^{32}$P]-labelled TS primer (Genasia Scientific Inc, Taipei, Taiwan), 0.1 µg CX primer (Genasia Scientific Inc, Taipei, Taiwan), 2 units of Taq DNA polymerase (HT Biotech Ltd, Taipei, Taiwan), 20 mM Tris-HCl (pH 8.3), 1.5 mM $MgCl_2$, 63 mM KCl, 0.005% Tween-20, 1 mM EGTA, 50 µM dNTPs, and 0.1 mg $ml^{-1}$ bovine serum albumin. The reaction mixture was incubated at 24°C for 15 min following PCR amplification of 30 cycles of 94°C for 30 s, 55°C for 30 s, and 72°C for 1 min in a DNA Thermal Cycler (Perkin Elmer Cetus, Norwalk, CT, USA). RNase digestion was performed as a control to confirm that the activity was that of telomerase. For these reactions, cell extracts were preincubated with 200 $\mu g\ ml^{-1}$ of RNase A (Boehringer Mannheim, Germany) at room temperature for 20 min before being added to the reaction mixture. The PCR products were resolved by electrophoresis on a nondenaturing 10% polyacrylamide gel (PAGE) in a buffer containing 54 mM Tris-HCl, pH 8.0, 54 mM boric acid, 1 mM EDTA. Positive telomerase activity in an extract is determined by the presence of six-nucleotide ladder of TRAP products in PAGE which are sensitive to RNase A treatment.

### Determining the level of telomerase activity

The autoradiograms of TRAP products on PAGE were quantitated by computerized scanning densitometry using ImageQuan (Molecular Dynamics, CA, USA) (Cheng et al, 1997). Relative levels of telomerase activity were measured by comparing the intensity of the ladder signals with serial dilution of the NPC076 cell line (3, 30 and 300 cells). Tissues with telomerase activity

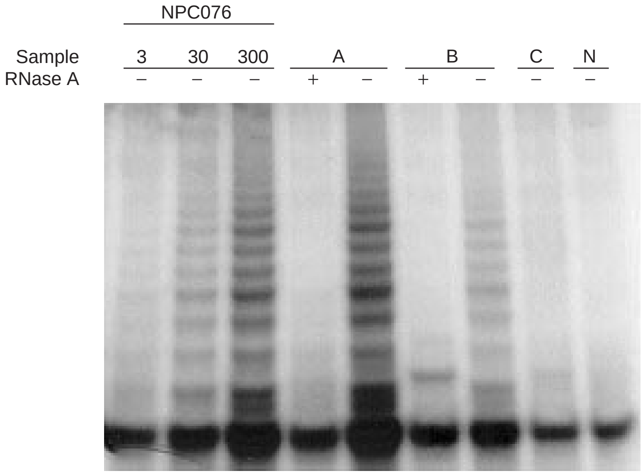

**Figure 1** Levels of telomerase activity in different nasopharyngeal tissues. Protein extracts from 3, 30, or 300 NPC076 cells were used as positive controls. Levels of telomerase activity from samples A, B, and C were compared with those of NPC076 cells, as described in the Materials and methods section. In sample A, level of telomerase activity was greater 300 NPC076 cells, and was considered as strong-positive. In sample B, level of telomerase activity was between 3 and 30 NPC076 cells, and was considered as weak-positive. In sample C, telomerase activity was less than 3 NPC076 cells and was considered as negative. Sample N was negative control, which included all assay components except protein extract.

*© 2000 Cancer Research Campaign* 

equivalent to less than three NPC076 cells were scored as 'undetectable' or 'negative'. Tissues with telomerase activity equivalent to 3–30 cells were scored 'low levels', while tissues with telomerase activity equivalent to 30–300 or greater than 300 NPC076 cells were scored as 'moderate' or 'high level', respectively. Examples of the results are shown in Figure 1.

### Statistical analysis

For statistical analysis, we divided all samples into three groups: inflammation or lymphoid hyperplasia (without metaplasia or carcinoma), squamous metaplasia (without carcinoma), and carcinoma tissues, according to the degree of pathological transformation. To examine how telomerase activity varied in different factors, Fisher's exact test was used due to small number of subjects studied. These factors included pathological findings (inflammation or hyperplasia, squamous metaplasia, carcinomas), presence or absence of hyperplasia (germinal centre, vessel, lymphoid, mucus cells), and tumour staging (T and N). All *P*-values were tow-sided. Statical significance was considered if $P \leq 0.05$. SAS/win 6.12 was used.

### RESULTS

### Telomerase activity in nasopharyngeal tissues

Examples of the results are shown in Figure 1. Each patient's conditions including histological examination data and the level of telomerase activity are shown on Table 1. Summary of the diagnosis is listed in Table 2. Positive telomerase activity was detected in 1 of 17 (5.9%) inflammatory or hyperplasia tissues, 3 of 8 (37.5%) samples with squamous metaplasia, and 25 of 29 (86.2%) carcinomas. Telomerase activity in these three groups was significantly different ($P < 0.001$), indicating that telomerase activity was strongly correlated with the degree of cellular transformation in nasopharyngeal tissues. For each tissue sample, the degree of telomerase activity was examined for correlation with commonly found pathological features, such as germinal centre hyperplasia, vessel hyperplasia, lymphoid hyperplasia, mucus cell hyperplasia, and metaplasia. Results are summarized in Table 3. There was no significant correlation of telomerase activity with any of the above hyperplastic features. However, telomerase activity was significantly associated with the presence of squamous metaplasia ($P < 0.001$).

### Telomerase activity in nasopharyngeal cancers

The clinicopathological variables and the level of telomerase activity from 29 NPC patients were further analysed. The correlation of telomerase activity with primary tumour stage or lymph node status is summarized in Table 4. Relative level of telomerase activity was determined by comparison with the activity of control cells (Materials and methods). Of the 29 carcinomas, four samples had no detectable telomerase activity, nine samples had a low level, ten a moderate level, and six a high level of telomerase activity. For the four NPC patients without detectable telomerase activity, two had T4 lesion, one had a T3 and one a T2 lesion (Table 1). Low levels of telomerase activity occurred mostly (8/9) in lower stages (T1–2) of NPC, and high levels mostly (5/6) in advanced stages (T3–4) (Table 4). We also found that telomerase activity was associated with tumor T stage ($P = 0.024$) but not with the status of neck lymph node metastatic ($P = 0.292$) (Table 4).

### DISCUSSION

Investigation of various tumours indicates that reactivation of telomerase activity may occur at different stages depending on the types of cancers. For example, reactivation of telomerase activity was found in benign or precancerous tissues of the breast (Sugino et al, 1996), liver (Tahara et al, 1995), head and neck (Mutirangura et al, 1996; Califano et al, 1996; Mao et al, 1996), thyroid gland (Cheng A-J et al, 1998) and colorectum (Tang et al, 1998). Moreover, some infective tissues were also found positive for telomerase activity (Tahara et al, 1995; Kameshima et al, 1999). In studies of prostate and gastric cancers, however, telomerase activity was only detected in malignant tissues (Hiyama et al, 1997; 1995). These results indicate that reactivation of telomerase activity during the development of cancers may occur at distinct carcinogenic steps, depending on the specific type of tissue involved. In our study, telomerase activity was not only found in carcinomas but also in some metaplastic tissues and a small percentage of inflammatory and hyperplastic tissues, suggesting the enzyme reactivation in NPC may occur at a pre-cancerous stage of carcinogenesis.

Cellular proliferation and transformation are different aspects of carcinogenesis, but both are required for malignant formation. Reports on the roles of telomerase activation with respect to these two processes are different in various types of cancers. For example, telomerase activity was correlated with a poorer grade of cellular differentiation but not with tumour size in liver cancer (Tahara et al, 1995), indicating that telomerase may be more involved in cellular transformation. On the other hand, telomerase activity has been found in mitotically active or regenerating somatic cells (Ramirez et al, 1997; Herle-Bachor et al, 1996; Kyo et al, 1997), suggesting that it is associated with cellular proliferation. Recently, studies in cell-line models further support the contention that telomerase expression is associated with cellular immortalization but not with transformation (Morales et al, 1999; Jiang et al, 1999). In this present study, we did not find a significant association of telomerase activity with hyperplasia per se in nasopharyngeal tissue, but rather with metaplastic change. The reason for this is unclear. However, all of the above indicate that telomerase may involve in multiple physiological and pathological functions.

Recently a number of laboratories have identified a putative telomerase repressor gene mapped to chromosome region 3p14–21 or 3p21–22 (Mehle et al, 1998; Cuthbert et al, 1999; Tanaka et al, 1998; Horikawa et al, 1998). For example, loss of heterozygosity at chromosome 3p correlated with telomerase activity in renal cell carcinoma (Mehle et al, 1998). Strong repression of telomerase was observed following transfer of chromosome 3 into breast cancer cells (Cuthbert et al, 1999). In nasopharyngeal cancer patients, loss of heterozygosity or homozygous deletion at the chromosome region of 3p14–21 was often found (Huang et al, 1994; 1995). Although the association of telomerase reactivation and chromosome deletion at 3p in NPC patients was not clear, with such coincidence of these two it may be postulated that deletion of the chromosome 3p may reactivate telomerase activity leading to acceleration of malignant transformation of nasopharyngeal lesions.

© 2000 Cancer Research Campaign

**Table 1** Pathohistological characteristics and telomerase activity of the NPC patients

| | | | Tumour stage | | | Histological examination[2] | | | | | | |
|---|---|---|---|---|---|---|---|---|---|---|---|---|
| **Patient[1]** | **Diagnosis** | **Telomeras[1]** | **T** | **N** | **M** | **Ca** | **Metapl** | **Gem Hp** | **Lym Hp** | **Mucus** | **Vel Hp** | **Sinus** |
| 1 | inflam/hyp | 0 | | | | 0 | 0 | 0 | 0 | 0 | 0 | 0 |
| 2 | inflam/hyp | 0 | | | | 0 | 0 | 0 | 0 | 0 | 0 | 0 |
| 3 | inflam/hyp | 0 | | | | 0 | 0 | 0 | 0 | 0 | 0 | 0 |
| 4 | inflam/hyp | 0 | | | | 0 | 0 | 0 | 1 | 1 | 0 | 0 |
| 5 | inflam/hyp | 0 | | | | 0 | 0 | 0 | 1 | 0 | 0 | 0 |
| 6 | inflam/hyp | 0 | | | | 0 | 0 | 0 | 0 | 0 | 0 | 0 |
| 7 | inflam/hyp | 0 | | | | 0 | 0 | 1 | 1 | 0 | 0 | 0 |
| 8 | inflam/hyp | 0 | | | | 0 | 0 | 0 | 0 | 0 | 0 | 0 |
| 9 | inflam/hyp | 0 | | | | 0 | 0 | 0 | 0 | 0 | 0 | 0 |
| 10 | inflam/hyp | 0 | | | | 0 | 0 | 0 | 1 | 1 | 0 | 0 |
| 11 | inflam/hyp | 0 | | | | 0 | 0 | 0 | 0 | 0 | 0 | 0 |
| 12 | inflam/hyp | 0 | | | | 0 | 0 | 1 | 0 | 0 | 0 | 0 |
| 13 | inflam/hyp | 0 | | | | 0 | 0 | 0 | 1 | 1 | 0 | 0 |
| 14 | inflam/hyp | 0 | | | | 0 | 0 | 1 | 0 | 0 | 0 | 0 |
| 15 | inflam/hyp | 0 | | | | 0 | 0 | 0 | 1 | 1 | 0 | 0 |
| 16 | inflam/hyp | 0 | | | | 0 | 0 | 0 | 0 | 0 | 0 | 0 |
| 17 | inflam/hyp | 2 | | | | 0 | 0 | 0 | 1 | 1 | 0 | 1 |
| 18 | metaplasia | 0 | | | | 0 | 1 | 0 | 1 | 0 | 0 | 0 |
| 19 | metaplasia | 0 | | | | 0 | 1 | 0 | 1 | 0 | 0 | 0 |
| 20 | metaplasia | 0 | | | | 0 | 1 | 1 | 1 | 0 | 1 | 0 |
| 21 | metaplasia | 0 | | | | 0 | 1 | 1 | 1 | 1 | 0 | 0 |
| 22 | metaplasia | 0 | | | | 0 | 1 | 0 | 1 | 0 | 0 | 0 |
| 23 | metaplasia | 1 | | | | 0 | 1 | 1 | 0 | 1 | 0 | 0 |
| 24 | metaplasia | 1 | | | | 0 | 1 | 1 | 1 | 0 | 0 | 0 |
| 25 | metaplasia | 3 | | | | 0 | 1 | 0 | 1 | 1 | 1 | 0 |
| 26 | cancerous | 0 | 2 | 2 | 0 | 1 | 0 | 0 | 1 | 0 | 0 | 0 |
| 27 | cancerous | 0 | 3 | 0 | 0 | 1 | 0 | 0 | 1 | 0 | 0 | 0 |
| 28 | cancerous | 0 | 4 | 0 | 0 | 1 | 0 | 0 | 1 | 0 | 0 | 0 |
| 29 | cancerous | 0 | 4 | 2 | 0 | 1 | 1 | 1 | 1 | 1 | 0 | 0 |
| 30 | cancerous | 1 | 1 | 1 | 0 | 1 | 1 | 1 | 1 | 0 | 0 | 0 |
| 31 | cancerous | 1 | 2 | 0 | 0 | 1 | 0 | 0 | 1 | 0 | 0 | 0 |
| 32 | cancerous | 1 | 1 | 1 | 0 | 1 | 0 | 0 | 1 | 0 | 0 | 0 |
| 33 | cancerous | 1 | 1 | 0 | 0 | 1 | 1 | 0 | 1 | 0 | 1 | 0 |
| 34 | cancerous | 1 | 1 | 1 | 0 | 1 | 1 | 0 | 1 | 0 | 0 | 0 |
| 35 | cancerous | 1 | 2 | 1 | 0 | 1 | 1 | 1 | 0 | 0 | 0 | 0 |
| 36 | cancerous | 1 | 2 | 0 | 0 | 1 | 1 | 1 | 1 | 0 | 0 | 0 |
| 37 | cancerous | 1 | 4 | 3 | 0 | 1 | 1 | 1 | 1 | 1 | 1 | 0 |
| 38 | cancerous | 1 | 1 | 1 | 0 | 1 | 1 | 0 | 1 | 1 | 0 | 0 |
| 39 | cancerous | 2 | 3 | 1 | 0 | 1 | 1 | 1 | 1 | 0 | 0 | 0 |
| 40 | cancerous | 2 | 2 | 3 | 0 | 1 | 1 | 1 | 1 | 1 | 0 | 0 |
| 41 | cancerous | 2 | 2 | 1 | 0 | 1 | 0 | 0 | 1 | 0 | 0 | 0 |
| 42 | cancerous | 2 | 1 | 1 | 0 | 1 | 0 | 0 | 1 | 0 | 0 | 0 |
| 43 | cancerous | 2 | 2 | 2 | 0 | 1 | 0 | 0 | 1 | 0 | 1 | 0 |
| 44 | cancerous | 2 | 3 | 1 | 1 | 1 | 1 | 0 | 0 | 1 | 0 | 0 |
| 45 | cancerous | 2 | 2 | 0 | 0 | 1 | 1 | 0 | 0 | 0 | 0 | 0 |
| 46 | cancerous | 2 | 4 | 3 | 0 | 1 | 0 | 0 | 1 | 0 | 0 | 0 |
| 47 | cancerous | 2 | 3 | 3 | 0 | 1 | 0 | 1 | 0 | 0 | 0 | 0 |
| 48 | cancerous | 2 | 1 | 1 | 0 | 1 | 0 | 0 | 1 | 0 | 0 | 0 |
| 49 | cancerous | 3 | 1 | 1 | 0 | 1 | 1 | 1 | 0 | 0 | 0 | 0 |
| 50 | cancerous | 3 | 4 | 2 | 0 | 1 | 0 | 0 | 1 | 0 | 0 | 0 |
| 51 | cancerous | 3 | 4 | 2 | 0 | 1 | 1 | 1 | 1 | 1 | 0 | 0 |
| 52 | cancerous | 3 | 3 | 0 | 0 | 1 | 1 | 0 | 1 | 0 | 0 | 0 |
| 53 | cancerous | 3 | 4 | 2 | 0 | 1 | 0 | 0 | 1 | 1 | 1 | 1 |
| 54 | cancerous | 3 | 4 | 1 | 0 | 1 | 0 | 0 | 1 | 0 | 0 | 0 |

[1] Telomerase activity was determined as negative (0), low (1), moderate (2), and high (3) levels; [2]histological examinations for cancer (Ca), metaplasia (Metapl), germinal centre hyperplasia (Gem Hp), lymphoid hyperplasia (Lym Hp), mucous cell hyperplasia (Mucus), vessel hyperplasia (Vel Hp) and sinus were indicated as either presence (1) or absence (0)

Telomerase activity in nasopharyngeal carcinoma tissues has been reported previously (Cheng RYS et al, 1998). Basically, we agree with previous findings that most malignant nasopharyngeal tissues are positive for telomerase activity. In this present study, we extend their observation to non-cancerous nasopharyngeal lesions by evaluating the enzyme activity in hyperplastic and metaplastic nasopharyngeal tissues. Percentages of the enzyme activity were found to increase from hyperplastic through metaplastic to malignant tissues. For the telomerase-positive lymphoid hyperplasia patient (patient 17), other pathological features such as mucus hyperplasia and sinusitis were also found. In this case, there is insufficient data to make any assumption about the mechanism

*© 2000 Cancer Research Campaign*

**Table 2** Telomerase activity in diseased nasopharyngeal tissues

| Pathology | *n* | Telomerase activity | | | |
|---|---|---|---|---|---|
| | | Negative (%) | Low (%) | Moderate (%) | High (%) |
| Inflammation or hyperplasia | 17 | 16 (94.1) | 0 (0%) | 1 (5.9) | 0 (0%) |
| Squamous metaplasia | 8 | 5 (62.5) | 2 (25.0) | 0 (0%) | 1 (12.5) |
| Carcinomas | 29 | 4 (13.8) | 9 (31) | 10 (34.5) | 6 (20.7) |

P < 0.001 (Fisher's exact test).

**Table 3** Correlation between telomerase activity and pathological variants of nasopharyngeal lesions

| Variable | Category | *n* | Telomerase activity | | | | |
|---|---|---|---|---|---|---|---|
| | | | Negative (%) | Low (%) | Moderate (%) | High (%) | *P* value |
| Germinal centre | no | 37 | 19 (51.4) | 5 (13.5) | 8 (21.6) | 5 (13.5) | 0.387 |
| hyperplasia | yes | 17 | 6 (35.3) | 6 (35.3) | 3 (17.6) | 2 (11.8) | |
| Vessel | no | 48 | 24 (50.0) | 9 (18.8) | 10 (20.8) | 5 (10.4) | 0.150 |
| hyperplasia | yes | 6 | 1 (16.7) | 2 (33.3) | 1 (16.7) | 2 (33.3) | |
| Lymphoid | no | 16 | 10 (62.5) | 2 (12.5) | 3 (18.8) | 1 (6.3) | 0.523 |
| hyperplasia | yes | 38 | 15 (39.5) | 9 (23.7) | 8 (21.1) | 6 (15.8) | |
| Mucous cell | no | 39 | 19 (48.7) | 8 (20.5) | 8 (20.5) | 4 (10.3) | 0.765 |
| hyperplasia | yes | 15 | 6 (40.0) | 3 (20.0) | 3 (20.0) | 3 (20.0) | |
| Squamous | no | 31 | 19 (61.3) | 2 (6.4) | 7 (22.6) | 3 (9.7) | < 0.001 |
| metaplasia | yes | 23 | 6 (21.6) | 9 (39.1) | 4 (17.4) | 4 (17.4) | |

Fisher's exact test was used.

**Table 4** Correlation between telomerase activity and pathological variants in nasopharyngeal carcinoma

| Variable | *n* | Telomerase activity | | | | |
|---|---|---|---|---|---|---|
| | | Negative (%) | Low (%) | Moderate (%) | High (%) | *P* value |
| T1–2 | 16 | 1 (6.3) | 8 (50.0) | 6 (37.5) | 1 (6.3) | 0.024 |
| T3–4 | 13 | 3 (23.1) | 1 (7.7) | 4 (30.8) | 5 (38.5) | |
| N0 | 7 | 2 (28.6) | 3 (42.8) | 1 (14.3) | 1 (14.3) | 0.292 |
| N1 | 12 | 0 (0) | 5 (41.7) | 5 (41.7) | 2 (16.6) | |
| N2–3 | 10 | 2 (20.0) | 1 (10.0) | 4 (40.0) | 3 (30.0) | |

Fisher's exact test was used.

of telomerase reactivation. Whether the other pathological feature or a combination of factors contributed to the reactivation of telomerase is being addressed. For the patients with telomerase-positive metaplastia, follow-up studies are being conducted to investigate whether the presence of telomerase activity may correlate with further malignant change. Although the relationship between squamous metaplasia and carcinomatous change in nasopharyngeal tissue is still unknown, the expression of telomerase activity in a subset of the patients with metaplasia may provide additional clues for us to examine. In carcinoma patients, detection of higher levels of telomerase activity in the majority of advance-staged disease (T3–T4) as compared to the early stages (T1–T2) ($P = 0.024$) suggests a positive association of telomerase activity with tumour progression. Prospective investigation of this question is underway, especially to evaluate whether telomerase activity correlates with cancer prognosis.

In view of the high frequency of telomerase activity in malignant tissues and in a subset of metaplastic tissues, telomerase may potentially be used for monitoring the pre-malignant change of nasopharyngeal lesions. Currently, the diagnosis rests on histopathological examination, as there is no useful molecular marker for detecting NPC. Serological anti-EBV antibodies provide only limited information, because over 90% of the Far East population have subclinical infections in early childhood, and are therefore seropositive. In conclusion, the present study suggests that telomerase activation may play a role in the development of nasopharyngeal cancer. Since telomerase activity is found in the majority of nasopharyngeal cancers and a subset of metaplasia, this enzyme may be served as a reference to monitoring the status of abnormal nasopharyngeal tissues.

### REFERENCES

Blackburn EH (1992) Telomerase. *Annu Rev Biochem* **61**: 113–129

Califano J, Ahrendt SA, Meininger G, Westra WH, Koch WM and Sidransky D (1996) Detection of telomerase activity in oral rinses from head and neck squamous cell carcinoma patients. *Cancer Res* **56**: 5720–5722

Campisi J (1997) The biology of replicative senescence. *Eur J Cancer* **33**: 703–309

Chang JT, See LC, Tang SG, Lee SP, Wang CC and Hong JH (1996) The role of brachytherapy in early-stage nasopharyngeal carcinoma. *Int J Radiation Oncol Biol Phys* **36**: 1019–1024

Cheng A-J, Liao S-K, Chow S-E, Chen J-K and Wang T-CV (1997) Differential inhibition of telomerase activity during induction of differentiation in hematopoietic, melanoma, and glioma cells in culture. *Biochem Biophys Res Commun* **237**: 438–444

Cheng A-J, Lin J-D, Chang T and Wang T-CV (1998) Telomerase activity in benign and malignant human thyroid tissues. *Br J Cancer* **77**: 2177–2180

*© 2000 Cancer Research Campaign*

Cheng A-J, Tang R, Wang J-Y, Chang JT and Wang T-C (1999) Polymerase chain reaction-based enzyme immunoassay for quantitation of telomerase activity: application to colorectal cancers. *Jpn J Cancer Res* **90**: 280–285

Cheng RYS, Yuen PW, Nicholls JM, Zheng Z, Wei W, Sham JST, Yang XH, Cao L, Huang DP and Tsao SW (1998) Telomerase activation in nasopharyngeal carcinomas. *Br J Cancer* **77**: 456–460

Choi PHK, Suen MWN, Path MRC, Huang DP, Lo KW and Lee J (1993) Nasopharyngeal carcinoma: genetic changes, Epstein-Barr virus infection, or both. *Cancer* **72**: 2873–2878

Cuthbert AP, Bond J, Trott DA, Gill S, Broni J, Marriott A, Khoudoli G, Parkinson EK, Cooper CS and Newbold RF (1999) Telomerase repressor sequences on chromosome 3 and induction of permanent growth arrest in human breast cancer cells. *J Natl Cancer Inst* **91**: 37–45

Fandi A and Cvitkovic E (1994) Nasopharyngeal cancer: epidemiology, staging, and treatment. *Semin Oncol* **21**: 376–381

Fandi A and Cvitkovic E (1995) Biology and treatment of nasopharyngeal cancer. *Curr Opin Oncol* **7**: 255–263

Feng BC and Liu KL (1989) A morphological study of stromal microvasculature of nasopharyngeal precancerous lesions. *Clin Med J (Engl)* **104**: 422–424

Hedinger C, Williams ED and Sobin LH (1989) The WHO histological classification of thyroid tumors: a commentary on the second edition. *Cancer* **63**: 908–911

Herle-Bachor C and Boukamp P (1996) Telomerase activity in the regenerative basal layer of the epidermis in human skin and in immortal and carcinoma-derived skin keratinocytes. *Proc Natl Acad Sci USA* **93**: 6476–6481

Hermanek P, Hutter RVP, Sobin LH, Wagner G and Wittekind Ch (eds) (1997) *UICC TNM Atlas*, 4th ed. Springer: New York

Hiyama E, Yoyama T, Tatsumoto N, Hiyama K, Imamura Y and Matsuura Y (1995) Telomerase activity in gastric cancer. *Cancer Res* **55**: 3258–3262

Hiyama E, Kodama T, Shinbara K, Iwao Y, Itoh M, Hiyama K, Shay JW, Matsuura Y and Yokoyama T (1997) Telomerase activity is detected in pancreatic cancer but not in benign tumors. *Cancer Res* **57**: 326–331

Horikawa I, Oshimura M and Barrett JC (1998) Repression of the telomerase catalytic subunit by a gene on human chromosome 3 that induces cellular senescence. *Mol Carcinog* **22**: 65–72

Huang DP, Lo KW, Choi PHK, Ng AYT, Tsao SY, Yiu GKC and Lee JCK (1991) Loss of heterozygosity on the short arm of chromosome 3 in nasopharyngeal carcinoma. *Cancer Genet Cytogenet* **54**: 91–99

Huang DP, Lo KW, van Hasselt CA, Woo JKS, Choi PHK, Leung SF, Cheung ST, Caims P, Sidransky D and Lee JCK (1994) A region of homozygous deletion on chromosome 9p21–22 in primary nasopharyngeal carcinoma. *Cancer Res* **54**: 4003–4006

Jiang XR, Jimenez G, Chang E, Frolkis M, Kusler B, Sage M, Beeche M, Bodnar AG, Wahl GM, Tlsty TD and Chiu CP (1999) Telomerase expression in human somatic cells does not induce changes associated with a transformed phenotype. *Nat Genet* **21**: 111–114

Kameshima H, Yagihashi A, Yajima T and Watanabe N (1999) Helicobacter pylori infection induces telomerase activity in premalignant lesions. *Am J Gastroenterol* **94**: 547

Kieserman SP and Stern J (1995) Malignant transformation of hasopharyngeal lymphoid hyperplasia. *Otolaryngol Head Neck Surg* **113**: 474–476

Kristensen S, Tverteras K, Friedmann I and Thomsen P (1989) Nasopharyngeal Warthin's tumor: a metaplastic lesion. *J Laryngol Otol* **103**: 616–619

Ku W-C, Cheng A-J and Wang T-C V (1997) Inhibition of telomerase activity by PKC inhibitors in human nasopharyngeal cancer cells in culture. *Biochem Biophys Res Commun* **241**: 730–736

Kyo S, Takakura M, Kohama T and Inoue M (1997) Telomerase activity in human endometrium. *Cancer Res* **57**: 610–614

Liebowitz D (1994) Nasopharyngeal carcinoma: the Epstein-Barr virus association. *Semin Oncol* **21**: 376–381

Lu S, Day NE, Degos L, Lepage V, Wang P-C, Chan S-H, Simons M, McKnight B, Easton D, Zeng Y and deThe G (1990) Linkage of a nasopharyngeal carcinoma susceptibility locus to the HLA region. *Nature* **346**: 470–471

Mao L, El-Naggar AK, Fan YH, Lee JS, Lippman SM, Kayser S, Lotan R and Hong WK (1996) Telomerase activity in head and neck squamous cell carcinoma and adjacent tissues. *Cancer Res* **56**: 5600–5604

Mehle C, Lindblom A, Ljungberg B, Stenling R and Roos G (1998) Loss of heterozygosity at chromosome 3p correlates with telomerase activity in renal cell carcinoma. *Int J Oncol* **13**: 289–295

Morales CP, Holt S, Ouellette M, Kaur KJ, Yan Y, Wilson KS, White MA, Wright WE and Shay JW (1999) Absence of cancer-associated changes in human fibroblasts immortalized with telomerase. *Nat Genet* **21**: 115–118

Morin GB (1997) The implications of telomerase biochemistry for human disease. *Eur J Cancer* **33**: 750–560

Mutirangura A, Supiyaphun P, Trirekapaii S, Sriuranpong V, Sakuntabhai A, Yenrudi S and Voravud N (1996) Telomerase activity in oral leukoplakia and head and neck squamous cell carcinoma. *Cancer Res* **6**: 3530–3533

Ramirez RD, Wright WE, Shay JW and Taylor RS (1997) Telomerase activity concentrates in the mitotically active segments of human hair follicles. *J Invest Dermatol* **108**: 113–117

Shay JW and Bacchetti S (1997) A survey of telomerase activity in human cancer. *Eur J Cancer* **33**: 787–791

Sugino T, Yoshida K, Bolodeokul J, Tahara H, Buley I, Manek S, Wells C, Goodison S, Ide T, Suzuki T, Tahara E and Tarin D (1996) Telomerase activity in human breast cancer and benign breast lesions: diagnostic applications in clinical specimens, including fine needle aspirates. *Int J Cancer* **69**: 301–306

Tahara H, Nakanislii T, Kitamoto M, Nakashio R, Shay JW, Tahara E, Kajiyama G and Ide T (1995) Telomerase activity in human liver tissues: comparison between chronic liver disease and hepatocellular carcinomas. *Cancer Res* **55**: 2734–2736

Tanaka H, Shimizu M, Horikawa I, Kugoh H, Yokota J, Barrett JC and Oshimura M (1998) Evidence for a putative telomerase repressor gene in the 3p14.2–p21.1 region. *Genes Chromosomes Cancer* **23**: 123–133

Tang R, Cheng AJ, Wang JY and Wang TC (1998) Close correlation between telomerase expression and adenomatous polyp progression in multistep colorectal carcinogenesis. *Cancer Res* **58**: 4052–4054

Wright WE, Piatyszek MA, Rainey WE, Byrd W and Shay JW (1996) Telomerase activity in human germline and embryonic tissues and cells. *Dev Genet* **18**: 173–179

Yeldandi AV, Kaufman DG and Reddy JK (1996) Cell injury and cellular adaptation. In *Anderson's Pathology* (10 edn) Damjanov I and Linder J (eds) pp 357–386. Mosby-Year Book, Inc: New York

Yu MC (1991) *Nasopharyngeal carcinoma: epidemiology and dietary factors*. Lyon, IARC Scientific Publication: France

Zong YS and Zheng WJ (1989) A morphologic and follow-up study on the nasopharyngeal lymphoid hyperplasia and its relation to the cancer. *Clin Med J (Engl)* **102**: 625–629

*© 2000 Cancer Research Campaign*
